# The Cumming's Patent

**Published:** 1872-04

**Authors:** C. A. Woodward, W. A. Bronson

**Affiliations:** Chairman.; Secretary.


### EDITORIAL DEPARTMENT.

---

*The Cumming's Patent.*—At a meeting of the Executive Committee of New York and Brooklyn, March 21st, 1872, to hear the report of the delegates to Washington, the following resolutions were adopted; and it was desired that copies should be sent to the dental journals for publication, as a tribute eminently due to the gentlemen mentioned:

*Resolved,* That the energy, ability, and integrity which have been shown by our counsel, Gen. John A. Foster, and Samuel J. Glassey, Esq., in the preparation and presentation of the case of B. E. Gardner, against the Cumming's patent, just argued in the U. S. Supreme Court at Washington, entitles them to this recognition of their services by the Committee, and merit this public acknowledgment of their untiring labor, and skilful management of the case.

*Resolved,* That the Committee recognize their obligations to the gentlemen of the Baltimore and Washington Committees, and to their counsel, Orville Horwitz, Esq., for the efficient support they have so promptly and cheerfully given to the measures which it is hoped will result in our speedy and final success.

C. A. WOODWARD, *Chairman.*

W. A. BRONSON, *Secretary.*

